# Insulators for 2D nanoelectronics: the gap to bridge

**DOI:** 10.1038/s41467-020-16640-8

**Published:** 2020-07-07

**Authors:** Yury Yu. Illarionov, Theresia Knobloch, Markus Jech, Mario Lanza, Deji Akinwande, Mikhail I. Vexler, Thomas Mueller, Max C. Lemme, Gianluca Fiori, Frank Schwierz, Tibor Grasser

**Affiliations:** 1 Institute for Microelectronics (TU Wien), Gusshausstrasse 27–29, 1040 Vienna, Austria; 2 Ioffe Physical-Technical Institute, Polytechnicheskaya 26, St-Petersburg, Russia 194021; 3 Institute of Functional Nano & Soft Materials (FUNSOM), Collaborative Innovation Center of Suzhou Nanoscience and Technology, Soochow University, 199 Ren-Ai Road, Building 910, 215123 Suzhou, China; 4 The University of Texas at Austin, 10100 Burnet Rd. 160, Austin, TX 78758 USA; 5 Institute for Photonics (TU Wien), Gusshausstrasse 27–29, 1040 Vienna, Austria; 6 AMO GmbH, Advanced Microelectronic Center Aachen (AMICA), Otto-Blumenthal-Str. 25, 52074 Aachen, Germany; 7 Chair of Electronic Devices, RWTH Aachen University, Otto-Blumenthal-Str. 2, 52074 Aachen, Germany; 8 Dipartimento di Ingegneria dell’Informazione, Università di Pisa, 56122 Pisa, Italy; 9 Institute for Micro- and Nanoelectronics, Technical University Ilmenau, PF 100565, 98684 Ilmenau, Germany

**Keywords:** Two-dimensional materials, Electronic devices

## Abstract

Nanoelectronic devices based on 2D materials are far from delivering their full theoretical performance potential due to the lack of scalable insulators. Amorphous oxides that work well in silicon technology have ill-defined interfaces with 2D materials and numerous defects, while 2D hexagonal boron nitride does not meet required dielectric specifications. The list of suitable alternative insulators is currently very limited. Thus, a radically different mindset with respect to suitable insulators for 2D technologies may be required. We review possible solution scenarios like the creation of clean interfaces, production of native oxides from 2D semiconductors and more intensive studies on crystalline insulators.

## Abstract (web summary)

The lack of scalable, high-quality insulators is a major problem hindering the progress on electronic devices built from 2D materials. Here, the authors review the current state-of-the-art and the future prospects of suitable insulators for 2D technologies.

## Introduction

The field effect transistor (FET) is the fundamental building block for information processing and storage^1^. The working principle of FETs consists of controlling the current flow along a conductive surface channel formed between source and drain electrodes when a voltage is applied to the gate electrode, which is separated from the channel by an insulating layer (dielectric). The performance of FETs strongly depends not only on the properties of the channel material (e.g. its carrier mobility), but also on the quality of the interface to the gate insulator and the overall properties of that insulator.

Although historically many investigations have concentrated on the channel material and its physical and electrical properties striving for high mobilities or wide bandgaps, at the end it has always been the insulator and its interface with the channel material which decided the technological feasibility of a particular channel material considered. Most importantly, with the notable exception of Si/SiO_2_ (and possibly SiC/SiO_2_^2^), it has been the striking absence of suitable insulating materials which prevented superior channel materials from entering the mass market. Ge^3^, III–V materials^4^ and GaN^5^ have all raised considerable expectations as channel semiconductors for high mobility transistors, but for all them finding a compatible dielectric to produce high performance transistors has appeared challenging: (i) Ge native oxide (GeO) is water-soluble and the use of other materials produces a lattice mismatch which results in a high density of defects. (ii) III–V materials use Schottky contacts to directly contact the channel^6^ which increases gate leakage currents. (iii) GaN results in a high density of defects with most adjacent dielectrics^5^.

The last decade has seen a frantic search for channel materials with higher mobilities than Si in ultrathin layers to keep up scaling according to Moore’s law. For example, in an ultrathin layer of 5 nm, as is required for channel lengths smaller than 20 nm^7^, the mobility of Si is reduced far below 100 cm^2^/Vs^8,9^. As an attempt to address this limitation, 2D semiconductors, such as MoS_2_^10–16^, other transition metal dichalcogenides (TMDs, e.g. MoSe_2_^17^, MoTe_2_^18^, WS_2_^19^, WSe_2_^20^) or black phosphorus (BP)^21–23^, have been recently demonstrated as channel materials in FETs.

At a first glance, 2D materials seem to allow the arbitrary stacking of different material layers using van der Waals attractive forces^24^. Theoretical calculations have predicted excellent properties for devices built from 2D materials^25^. Also, considerable progress has been made in addressing fabrication-related issues^26,27^ and tuning electrical figures of merit, such as carrier mobility^16,28^ and on/off current ratios^14,29^. However, published 2D devices often suffer from non-competitive carrier mobilities, subthreshold swings (SS) and drifts of important device parameters (e.g. the threshold voltage shift over time), which may have nothing to do with 2D semiconductors, but arise from the gate insulators used. As a result, there is still no commercially competitive 2D transistor technology available today.

In this review we will discuss the current state-of-the-art regarding gate insulators for 2D technologies and discuss strategies for further improvements of the performance of 2D devices by using more suitable material combinations. While the main focus is on standard 2D FETs, we also note that the problems discussed here directly transfer to alternative device technologies, such as tunnel FETs^30^, ferroelectric FETs^31^, negative-capacitance transistors^32,33^ and analog field-effect devices (e.g. electro-optical modulators^34^, photodetectors^35^, and biosensors^36^), as all these devices require good insulating materials.

## State-of-the-art of 2D electronics

**Fig. 1:**
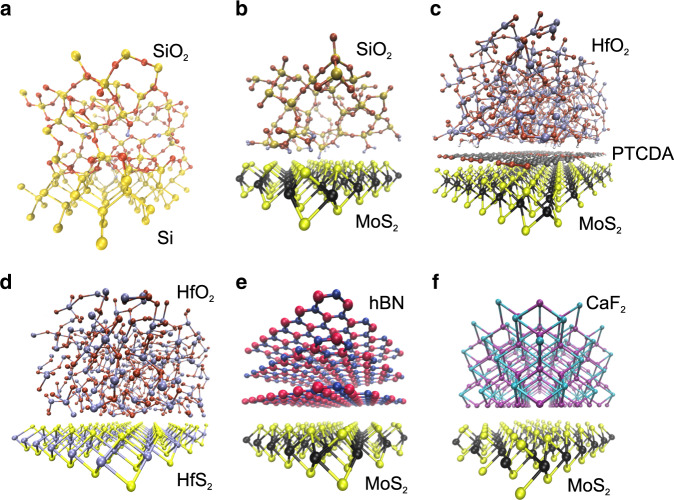
**Schematic channel/insulator interfaces in different device technologies**. **a** In 3D technologies an amorphous interface is formed between channel and insulator (example: Si/SiO_2_). **b** 3D insulators have poorly defined surfaces to form interfaces with the 2D channel (example: MoS_2_/SiO_2_). **c** The use of molecular crystal seeding layers improves the interface quality (example: MoS_2_/PTCDA/HfO_2_). **d** Oxidized 2D materials result in native oxides with good quality interfaces (example: HfS_2_/HfO_2_). **e** Van der Waals interface between crystalline 2D insulators and 2D channels (example: MoS_2_/hBN). **f** Ionic crystals with dangling bond-free inert surfaces and van der Waals bonded interface to a 2D material (example: MoS_2_/CaF_2_).

The core element of the FET is the combined system of semiconducting channel to gate insulator. Figure 1 schematically summarizes some examples of different channel/insulator configurations previously used. In Si technologies (Fig. 1a) the Si/SiO_2_ interface is excellent, particularly after the passivation of the about 2 × 10^12^ cm^−2^ Si dangling bonds at the interface using a forming gas (*H*_2_/*N*_2_) anneal, which reduces this number well below 10^10^ cm^−2^. Since currently no other competitive interface is available, oxides with higher dielectric constant k (high-k insulators) like Al_2_O_3_ and HfO_2_ typically require the use of a thin (<1 nm) SiO_2_ buffer layer. As for 2D devices, 3D oxides known from Si technologies result in a large number of dangling bonds at the 2D/3D interface (Fig. 1b)^37^. To passivate these imperfections, insulators and interfaces have been subjected to various annealing steps to reduce their defectivity, e.g. by the use of rapid thermal annealing (RTA)^38^. However, the resulting density of dangling bonds is still too high and deteriorates the device performance.

An alternative way to improve the interface between 2D semiconductors and 3D oxides (Fig. 1c) is the use of molecular crystal seeding layers (e.g. perylene-tetracarboxylic dianhydride (PTCDA)) when growing oxides using atomic layer deposition (ALD)^39,40^. However, these layers are formed by discrete molecules and thus making homogeneous films may be challenging. Furthermore, even if the use of molecular crystals improved the interface quality, we argue that typical monolayer thicknesses of three angstroms are not sufficient to completely block charge trapping by oxide defects. Another option is the partial oxidation of 2D materials which transforms them into their native oxides within the same heterostructure (Fig. 1d)^41–44^. It has been suggested that this process will lead to atomically abrupt and defect-free interfaces, which possibly might be as good as or even better than the Si/SiO_2_ interface (Fig. 1a).

Finally, crystalline insulators like layered 2D insulators such as hexagonal boron nitride^45^ (hBN, Fig. 1e) or ionic crystals like calcium fluoride^46^ (CaF_2_, Fig. 1f) have been used. The surfaces of these materials are chemically inert and free of dangling bonds. This results in well-defined van der Waals interfaces with 2D materials^47^, which is a considerable advantage of crystalline insulators over 3D oxides.

Among the possible insulators discussed above, the most promising are those which will be scalable down to equivalent oxide thicknesses (EOT, i.e. the thickness of SiO_2_ which would produce the same capacitance as the insulator in use) below 1 nm, as required for channel lengths below 10 nm, as well as those manufacturable with typical semiconductor process technology. In order to achieve high device performance, the insulators need to meet stringent requirements regarding (i) low gate leakage currents^48^ (<10^−2^ A/cm^2^), (ii) low density of interface traps^40^ (*D*_it_ < 10^10^ cm^−2^ eV^−1^), (iii) low density of border traps in the gate insulator^49^ (*D*_ot_ < 10^17^ cm^−3^ eV^−1^ for active traps^50^), and (iv) high dielectric strength (>10 MV/cm).

**Fig. 2:**
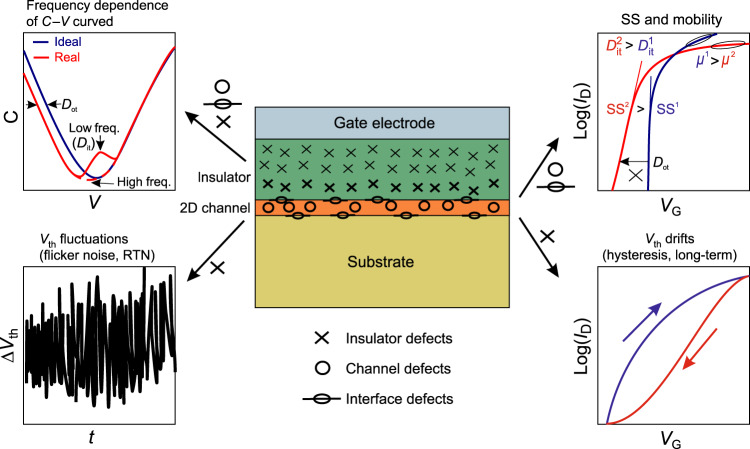
**Commonly measured effects caused by different defects in a 2D device**. C–V characteristics measured at different frequencies contain information about *D*_it_ (humps at low frequencies) and *D*_ot_ (stretch-out). If defect density is extracted from 1/*f* noise measurements, it will mostly consist of contributions from border defects (*D*_ot_), while channel defects and interface states (*D*_it_) are typically too fast. *D*_it_, on the other hand, degrades *SS* and mobility. *D*_ot_ causes instabilities of *V*_th_, such as hysteresis and long-term drifts. Insulator defects far from the interface are slow and thus mostly lead to a permanent shift of the threshold voltage.

  Figure 2 discusses some commonly measured effects in 2D devices which can be attributed to defects in the channel, in the insulator and at their interface. For instance, fast defects located at the interface (e.g. oxide dangling bonds) and in the channel (e.g. sulfur vacancies in MoS_2_) typically contribute to *D*_it_, which can be extracted from capacitance–voltage (C–V) measurements at different frequencies. Fast charge exchange between these defects and the channel also affects SS, while scattering at these defects degrades the mobility. Slow border traps are typically situated in the insulator within a few nanometers from the interface. They lead to various instabilities of the device threshold voltage, such as flicker (1/*f*) noise^51,52^, hysteresis^53,54^, and long-term drifts known from Si technologies as bias-temperature instabilities (BTI)^55^.

In the following we will discuss the requirements defined above for gate insulators in 2D electronics in more detail. We will also touch upon the impact of mechanical strain effects on the properties of thin insulators, which become important for flexible electronics applications.

### Gate leakage current

Aggressive scaling of the gate insulator increases direct tunneling^56,57^ and thus results in large leakage currents already at low voltages. In addition to direct tunneling through the insulator, Fowler-Nordheim tunneling through the bent barrier and trap-assisted tunneling (TAT), which dominates if the insulator contains a significant number of defects, become performance limiting factors.

**Fig. 3:**
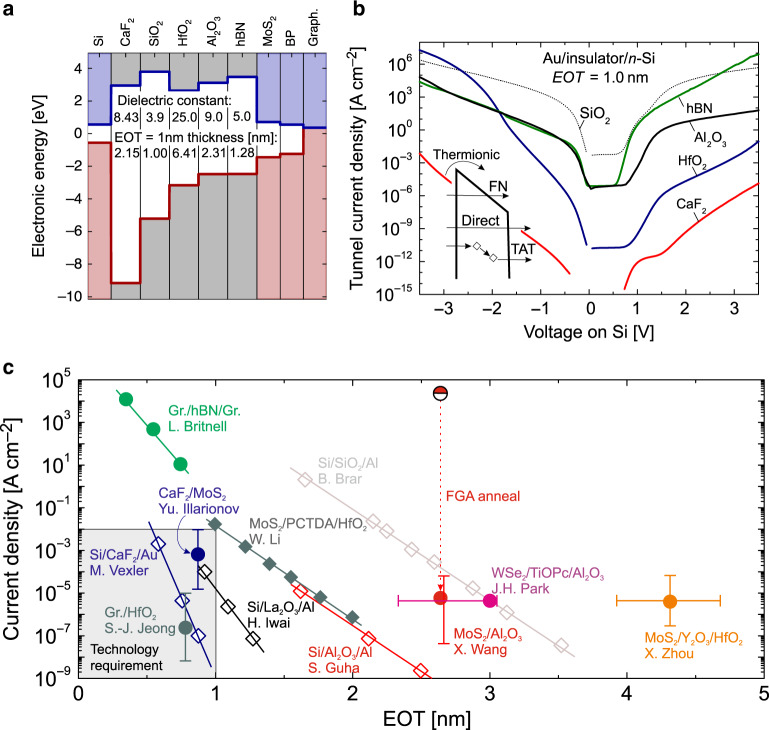
**Gate leakage currents through different insulators**. **a** Band diagram showing the alignment of the band gaps of some previously used insulators in 2D FETs relative to Si and typical 2D channel materials. **b** The leakage currents through the metal-insulator-semiconductor structures with these insulators for an EOT of 1 nm calculated using the WKB approach^57^ considering direct, FN tunneling and thermionic emission. The inset shows the important contributions to the tunneling current. For defective oxides, trap-assisted tunneling can lead to a significant contribution at low voltages, which is not accounted for in our best-case model. **c** Experimental gate leakage currents versus EOT measured at standard FET operating gate voltages 1–3 V. Literature data shown with open symbols for Si-based^59–62^ and filled symbols for 2D-based structures^39,40,46,63–66^.

Up to now, a variety of insulators have been already investigated for 2D FETs. The most widely used are thermally grown SiO_2_^13,20,58^ as a substrate/back-gate, and conventional high-k oxides such as Al_2_O_3_^16^ and HfO_2_^10,38^ for top-gated structures. In addition, the 2D crystalline insulator hBN^11,45,58^, as well as the crystalline CaF_2_^46^ have been used. The electric parameters of these insulators at a physical thickness of 1 nm EOT are summarized in the band diagram in Fig. 3a. The common understanding is that the most promising materials for scaling would be those with wider bandgaps and larger dielectric constants, i.e. high-k oxides. Indeed, modeling results show that these insulators can lead to considerably smaller leakage currents if fabricated with sufficient quality and a minimum number of defects (Fig. 3b). In Fig. 3c we compare the experimental values for the gate leakage currents in test structures and complete devices from Si^59–62^ and 2D^39,46,63–66^ technologies. In agreement with theoretical predictions, the lowest gate leakage currents have been obtained for HfO_2_^65^, which has the highest permittivity of 25, and for CaF_2_^46,62^, which is a crystalline and thus mostly defect-free insulator with a bandgap of 12.1 eV. Also, we note that epitaxial oxides like La_2_O_3_ or Gd_2_O_3_ have been previously considered for applications in Si devices^61,67^, and they may be a promising option for scaled 2D FETs as well. At present, leakage currents through amorphous oxides on 2D materials strongly depend on the material quality, perhaps more than on their nominal properties. When looking at Al_2_O_3_ data, we see that the gate leakage currents can be dramatically reduced if the insulator quality is improved by using a forming gas anneal (FGA)^66^. Nevertheless, the best values are still far from those measured for the same insulator in Si technologies^60^. The same issues may occur for native oxides of 2D materials, which are not well studied but can be expected to be similar to conventional high-k oxides in terms of structure and properties.

The layered 2D insulator hBN shows extremely high leakage currents for sub-1 nm EOT^63^, which is due to its rather narrow bandgap and low permittivity, and in agreement with modeling data. At this point it is worth noting that the nature of the leakage current across multilayer 2D dielectrics is not well understood because many new factors not present in traditional 3D dielectrics may play a significant role. Among them, the most important are: (i) plane-to-plane interactions and electron tunneling across van der Waals structures; (ii) synthesis process dependence (i.e. different density of native defects); (iii) confinement of the leakage current at local defects^68^; and (iv) dependence of the leakage current on the adjacent metallic electrode^68^. We finally note that the natural van der Waals gap between the insulator and the 2D material can also play an important role in reducing the tunneling current^69^. However, this mechanism has not been understood in full detail, and therefore deserves further investigations.

### Interface quality and device performance

In contrast to Si technologies, where covalent atomic bonds have to be accommodated at the interface, in 2D devices the quality of the insulator surface determines the quality of the interface. As schematically shown in Fig. 1, amorphous oxides have poorly defined surfaces with dangling bonds, especially when they are grown in thin layers. Thus, the use of crystalline insulators and native oxides with clean surfaces, as well as the passivation of amorphous interfaces using crystalline seeding layers is now considered a promising alternative.

In the literature the interface quality is often evaluated using the density of interface states *D*_it_. This quantity is linked to the device subthreshold swing as

1 $${{SS}}={\mathrm{ln}}\,(10)\frac{{k}_{{\rm{B}}}T}{q}\left(1+\frac{{C}_{{\rm{ch}}}+q{D}_{{\rm{it}}}}{{C}_{{\rm{ins}}}}\right)$$

where *C*_ins_ is the insulator capacitance and *C*_ch_ is the depletion layer capacitance in conventional FETs which becomes the channel capacitance in 2D FETs. Thus, scaling of EOT, which is inversely proportional to ${C}_{{\rm{ins}}}={k}_{{{\rm{SiO}}}_{2}}/{\rm{EOT}}$, should approach *SS* to a nearly ideal value of 60 mV/dec (at room temperature) for small *D*_it_. With increasing *D*_it_, fast charge trapping increases SS^70,71^. *D*_it_ can be either due to defects at the insulator surface, such as oxide dangling bonds, or channel defects (Fig. 2) which are typically the dominant contribution in devices with non-optimized channels. In addition to *D*_it_, *SS* can also be affected by Schottky barriers between the channel and source/drain electrodes^72^. However, strongly scaled insulators enhance the gate control over the channel potential, thereby reducing the impact of the Schottky barriers^46^.

**Fig. 4:**
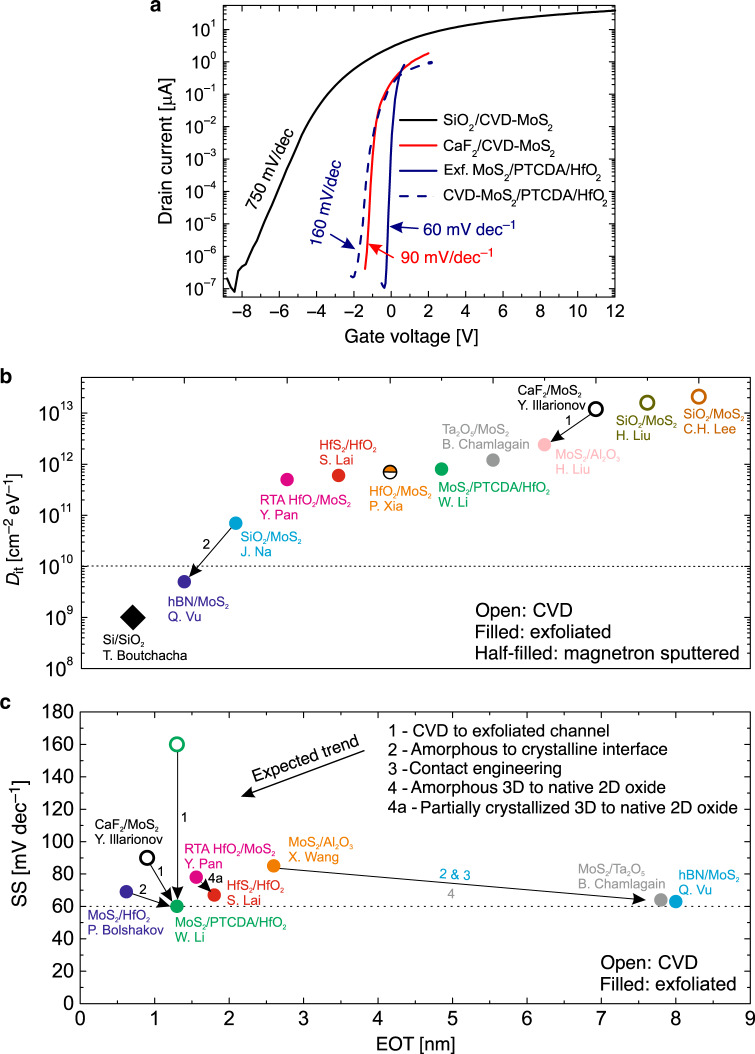
**Impact of the interface quality on the performance of 2D FETs**. **a** Gate transfer characteristics of CVD-grown MoS_2_ FETs with 25 nm SiO_2_^14^, 0.9 nm EOT CaF_2_^46^, 1.3 nm EOT PTCDA/HfO_2_^40^ and exfoliated devices of the latter technology^40^. **b** Comparison of *D*_it_ values measured for Si devices^78^ and different 2D technologies^38,40,41,43,46,54,73–77^. **c** Comparison of SS values for different 2D devices^16,38,40,41,43,46,54,66^ with EOT below 10 nm. For scaled insulators SS appears insensitive to further EOT scaling and mostly affected by the interface quality, which can be improved via the routes 1–4a. “Insulator/2D semiconductor” is for back-gated and “2D semiconductor/insulator” is for top-gated device configurations.

In Fig. 4a we compare typical transfer (*I*_D_–*V*_G_) characteristics measured for MoS_2_ FETs of different technologies^14,40,46^. Scaling EOT from 25 nm to 0.9–1.3 nm dramatically improves SS for all devices. However, a near-ideal SS of about 60 mV/dec has been obtained only for devices with exfoliated MoS_2_ using 1.3 nm EOT PTCDA/HfO_2_ insulators^40^, which is due to both the passivated interface of HfO_2_ by PTCDA and the low amount of channel defects in the exfoliated flakes. When using MoS_2_ channels grown by chemical vapour deposition (CVD) instead of exfoliated layers in otherwise identical devices, SS increases from 60 to 160 mV/dec^40^. Furthermore, MoS_2_ FETs with CVD-grown channels and 0.9 nm EOT CaF_2_ films^46^ also exhibited a SS of about 90 mV/dec. Thus, we argue that at the present level of 2D device technology, extracting *D*_it_ is not sufficient to assess the quality of the insulator surface in devices with CVD-grown channels, as *D*_it_ will be dominated by channel defects as well as adsorbates if the channel is not protected. In Fig. 4b we compare *D*_it_ values from literature (for more details see Box 1). Indeed, for all devices with CVD-grown channels^46,73,74^ *D*_it_ is close to 10^13^ cm^−2^ eV^−1^ compared to the 2 × 10^12^ cm^−2^ eV^−1^ of the *u*npassivated Si(100)/SiO_2_ interface which is reduced to below 10^10^ cm^−2^ eV^−1^ after annealing. *D*_it_ is further barely dependent on the gate insulator, which means that the quality of CVD samples must be improved including the optimization or avoidance of the transfer of grown films from other substrates to produce defect-free channels. Alternatively, other growth techniques of 2D materials may be introduced, such as magnetron sputtering^75^. However, most *D*_it_ values have been extracted from 2D FETs with exfoliated channels^38,40,41,43,54,76,77^, which typically contain a lower amount of channel defects and thus currently allow drawing better conclusions on the contribution of the insulator to the interface quality.

Still, most frequently extracted *D*_it_ values in exfoliated 2D FETs are still high and range from 5 × 10^11^ to 10^12^ cm^−2^ eV^−1^ for both conventional high-k oxides grown by ALD^38,40,76^ and native oxides of 2D materials^41,43^ obtained by oxidation. While the best *D*_it_ values achieved for 2D devices with SiO_2_ are two orders of magnitude lower^77^ than that, one can expect that further improvements will be achieved for high-k oxides by optimizing the deposition processes. However, without proper passivation of interfaces^40^ it still appears challenging to reach values below 10^10^ cm^−2^ eV^−1^, since oxide dangling bonds cannot be removed completely and the precise control of amorphous surfaces is difficult. As for native oxides of 2D materials, this research is at an early stage and thus further process optimization is required to achieve the desired improvement of *D*_it_. Consequently, the use of crystalline materials with well-defined and chemically inert van der Waals interfaces with 2D materials appears to be an ideal way forward. For instance, a *D*_it_ of 5 × 10^9^ cm^−2^ eV^−1^ achieved for hBN/MoS_2_ devices^54^ is already comparable to Si technologies^78^.

In Fig. 4c we compare SS from literature for different 2D FETs with scaled gate insulators^16,38,40,41,43,46,54,66^. Although from Eq. (1) one would expect the best SS for devices with smallest EOT, in reality the situation is more complex. For instance, the use of crystalline 2D insulators^54^ or native 2D oxides^41^ coupled with contact engineering to reduce the Schottky barriers leads to a near-ideal SS already for an EOT of ~8 nm, and even for thicker hBN layers in WSe_2_ FETs if transferred via contacts and clean van der Waals integration processes are used^79^. Again, this currently appears to be possible only with exfoliated channels, while for devices with CVD-grown films SS can be far from its ideal values even for EOT close to 1 nm^40,46^. We conclude that the use of high-quality channels and insulators with well-defined surfaces or native interfaces is the most important requirement to reduce *D*_it_ and achieve near-ideal SS for 2D FETs with sub-1 nm EOT gate insulators. The latter can also include partially crystallized amorphous oxides, since their surface quality has been shown to considerably improve after annealing at high temperatures^38^.

In addition to their impact on SS, *D*_it_ also manifests as charged defects, which lead to scattering of carriers and in turn dramatically reduces their mobility. For instance, graphene on an SiO_2_ surface with its numerous dangling bonds has a mobility of between 1000  and  25,000 cm^2^/Vs^80–82^, which increases to 27,000–65,000 cm^2^/Vs^82^ on hBN substrates. Subsequently, hBN has been also shown to improve the mobility in MoS_2_ FETs^11^. Ionic crystals, such as CaF_2_ can also yield high-quality interfaces with 2D materials^47,83,84^ which should result in improved mobilities.

Box 1 Details on *D*_it_ extraction It is commonly known that *D*_it_ values depend on both insulator and channel quality, while currently being lower for devices with exfoliated channels and/or crystalline insulators. However, when comparing *D*_it_ values provided in different literature reports it is important to understand that these values depend strongly on the extraction technique. For instance, *D*_it_ extracted from C–V measurements is typically due to fast interface defects only when the measurements are taken at high frequencies (MHz), otherwise border traps can also contribute. On the other hand, 1/*f* noise will be dominantly due to border defects. Furthermore, the use of the SS equation and other physics-based models may lead to some uncertainty since the impact of Schottky barriers is neglected. In the table in Box 1 we provide some details about devices and *D*_it_ extraction methods used in various literature reports^38,40,41,43,46,54,73–78^.

### Border traps and device stability

Contrary to interface states, border traps^49^ are situated at a certain distance from the interface which allows charge exchange with the channel through tunneling processes. While interface states are typically very fast, border traps are much slower, with their capture (*τ*_c_) and emission (*τ*_e_) time constants depending on the distance from the interface and structural relaxation following charge trapping. The best studied insulators are SiO_2_ and HfO_2_, which contain intrinsic (mostly oxygen vacancies and trapping sites at strained bonds) as well as extrinsic (for instance caused by trapped hydrogen atoms) defects. As confirmed on both Si^85^ and 2D^86,87^ devices, the time constants of border traps in amorphous oxides are widely distributed (from below nanoseconds to many years), which is a fundamental property of amorphous materials. As for other insulators, the physical and chemical nature of the prevalent defects is much less understood. For instance, the possible intrinsic defects in hBN identified using theoretical methods^88,89^ are nitrogen and boron vacancies and anti-sites. However, it appears that in crystalline insulators the surrounding of each particular defect is much more uniform and regular which should lead to a much narrower distribution of the time constants. Recently this was confirmed by comparing the hysteresis dynamics in MoS_2_ FETs with SiO_2_ and hBN, as in the latter case the hysteresis width starts to decrease when using slow sweeps^58^.

**Fig. 5:**
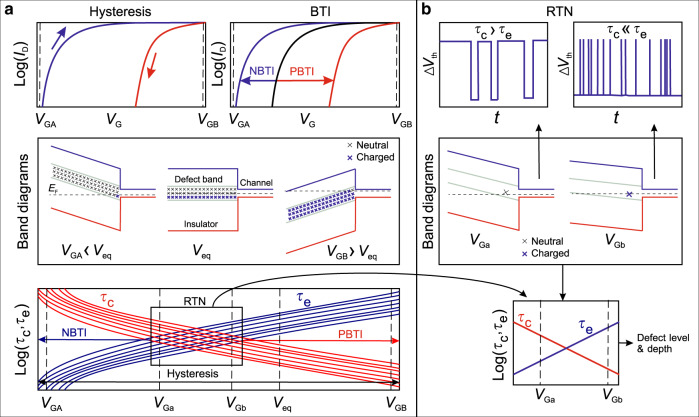
**Basic concept of charge trapping by insulator defects in 2D FETs**. **a** Schematics of hysteresis and BTI in large-area 2D n-FETs, assuming one acceptor-type defect band in the insulator. The applied *V*_G_ changes the number of charged defects by moving trap levels across the Fermi level, which changes not only the occupancy of the defects but also their time constants *τ*_c_ and *τ*_e_. A negative *V*_G_ brings more defects above the Fermi level and makes them neutral. This leads either to NBTI degradation after long stress or more negative *V*_th_ in the beginning of the forward *I*_D_–*V*_G_ sweep. A positive *V*_G_ brings more defects below *E*_F_ and makes them negatively charged, thus causing PBTI degradation or more positive *V*_th_ in the end of the forward sweep and during the reverse *I*_D_-*V*_G_ sweep. Thus BTI and hysteresis are both the result of an ensemble of defects with distributed *τ*_c_(*V*_G_) and *τ*_e_(*V*_G_). **b** Schematics of RTN in nanoscale 2D FETs, assuming one discrete defect in the channel. RTN is observed within a relatively narrow *V*_G_ range when *E*_F_ is close to the defect level. At more negative *V*_G_ the defect is slightly above *E*_F_ and thus mostly neutral. At more positive *V*_G_ it is slightly below *E*_F_ and thus mostly charged. Analysis of RTN traces measured at different *V*_G_ and *T* allows to extract the *τ*_c_(*V*_G_, *T*) and *τ*_e_(*V*_G_, *T*) dependences, which contain information about energy level, depth in the insulator, and relaxation energy of the defect.

Since border traps can capture and emit carriers, they can cause various instabilities in the device characteristics (Fig. 5). The most widely observed issues in 2D devices are the hysteresis of the gate transfer characteristics^11,12,45,54,58^ and long-term drifts of the threshold voltage^90–92^, which are commonly known from Si technologies as BTI, given their strong bias and temperature dependence^85^. Recent analysis of experimental results for MoS_2_^58,70,71,93,94^ and black phosphorus^23,50^ using non-radiative multiphonon (NMP) models^95^ (see more details in Box 2) suggests that hysteresis and BTI have the same microscopic origin and result from changes in the charge state of border traps (Fig. 5a). For instance, BTI degradation appears as a shift of the *I*_D_–*V*_G_ characteristics after application of some gate bias stress *V*_G_ for a certain stress time *t*_s_, which tends to recover when the stress is removed. Depending on the polarity of the applied *V*_G_ during stress, the phenomenon is then referred to as either positive (PBTI) or negative (NBTI) and may result in different shifts of the *I*_D_–*V*_G_ characteristics. Similarly, the hysteresis is a superposition of cumulative NBTI and PBTI shifts of *V*_th_ caused by charge trapping during *I*_D_–*V*_G_ sweeps. As a result, there is a difference in *V*_th_ when measuring forward and reverse sweeps. Since border defects have widely distributed capture and emission times, the faster ones contribute to the hysteresis and the slower ones also to BTI. In particular, the strong gate bias dependence of the time constants^71^, which is typical for border traps, results in a sizeable hysteresis. Other issues caused by border traps include flicker (1/*f*) noise^17,52^, which appears as random fluctuations of *I*_D_ (or, equivalently, *V*_th_) and for nanoscale devices decomposes into discrete steps known as random telegraph noise (RTN)^94,96^, as well as hot-carrier degradation (HCD) during device operation at non-zero drain bias, which is also known from Si technologies^97^ and has been already observed for 2D devices^98^.

In Si technologies these instabilities are commonly referred to as reliability issues, since they have a pronounced impact on the device performance only after many hours or weeks of operation. However, in 2D devices the typical densities of border traps can be orders of magnitude larger. As a result, the impact of defects on the device performance is already noticable at time zero. In other words, like in SiC, GaN, and other III–V devices, charge trapping related issues become a stability problem. Also, it has to be kept in mind that it is not the actual number of defects present in an insulator which determines device stability, but rather the number of active defects, that is, those defects which can change their charge state during device operation.

The most important aspect which determines the intensity of charge trapping by border traps, and consequently the effective density of active defects *D*_ot_ and the magnitude of the hysteresis and BTI, is their energetic alignment. In amorphous oxides, the defects are energetically aligned within certain defect bands which also have a sizeable width^99,100^. These defect bands are broadened if the surroundings of each defect varies, which is typical for amorphous materials. In the simplest case, defects can be either donor- (neutral or positive) or acceptor-like (neutral or negative). For donor-like states, if their thermodynamic trap level *E*_T_ is above the Fermi level *E*_F_, they are unoccupied and thus positively charged, and neutral otherwise. For acceptor-like states, on the other hand, defects are neutral for *E*_T_ > *E*_F_ and negatively charged otherwise. In contrast to amorphous oxides, defects in crystalline insulators are expected to form much narrower defect bands (or even discrete defect levels) as has already been shown for hBN^101^. Note that the density of active defects is always smaller if the defect bands are energetically far from the conduction (for n-FETs) and valence (for p-FETs) bands of the channel, which can be considered a design option for 2D FETs, as discussed below.

The density of insulator defects within a certain defect band may depend on the material type, deposition technique, stoichiometry, and annealing conditions. However, neither in 3D nor in 2D devices with amorphous oxides can the density of border traps be minimized towards undetectable levels, and thus these defects will be present even in perfectly optimized devices. We expect this to also hold for devices with native oxides of 2D materials, which ideally should have lower *D*_it_ compared to standard oxides while still having a comparable density of border traps within their characteristic defect bands. In contrast, in 2D FETs with crystalline insulators the defect densities inside narrow defect bands are expected to be significantly reduced.

Independently of the insulator used, the total number of defects is proportional to the channel area. Thus, as the device dimensions are scaled down to sub-100 nm and higher quality insulators are used, only a few defects per channel will remain even for amorphous oxides. However, the impact of each particular defect is inversely proportional to the channel area and becomes stronger for scaled devices^102^. As a result, in nanoscale 2D FETs capture and emission of a single carrier can strongly perturb the electrostatics inside the channel and thus cause RTN fluctuations in *I*_D_ and *V*_th_. A few recent studies for MoS_2_ FETs^94,96^ have already established that in general the dynamics of RTN in 2D FETs are very similar to Si technologies^103,104^. For instance, it was demonstrated that charge trapping events causing RTN are the same as those responsible for the hysteresis and BTI^94^, with the unique characteristic of border traps being the exponential *V*_G_ dependence of the time constants (Fig. 5b).

Box 2 Details on non-radiative multiphonon models The key feature of NMP models^95,158,159^ is accounting for the structural relaxation of the insulator defects following a charge capture or emission event. The structural relaxation is typically described assuming parabolic adiabatic potentials along a dominant reaction coordinate. It is very important to emphasize that due to structural relaxation upon charge exchange with the substrate (or gate), all time constants *τ*_c_ and *τ*_e_ are thermally activated and become shorter at higher temperatures. Also, all time constants are considerably larger than one would expect from tunneling theory alone, and even in thin insulators charges can be trapped for years at room temperature.

The figure below schematically illustrates charge trapping in the NMP model. The band and trap states are typically modeled as parabolic energy surfaces. Even if the trap energy is lower than the band energy, to first order the charge carrier has to overcome a barrier given by the intersection of the parabolas to change their state (in the classical limit^160^). By changing the electric field, the energy levels can be shifted with respect to each other. This leads to a change of barriers as seen by the defects, and thus the time it takes to change states depending on the temperature. Using the NMP model, charge exchange and temperature dependence can be modeled more accurately. The insets show the atomistic structure of a Hydrogen E’ center, which is one of the most likely defect candidate in SiO_2_, in the states 1 (neutral) and 2 (positive).

### Dielectric strength and breakdown mechanisms

The dielectric strength is characterized by two main parameters. The first one is the breakdown field *E*_BD_, which is the electric field at which a complete failure of the insulator takes place. This typically depends on the material type, quality and stoichiometry^105^, and ideally should exceed 10 MV/cm for EOT below 1 nm. *E*_BD_ can be obtained by applying current-voltage sweeps and extracting the voltage at which the leakage currents experience a large and irreversible increase of several orders of magnitude. The second parameter is the time to reach the time-dependent dielectric breakdown (TDDB)^106^ when the dielectric is exposed to a constant voltage stress.

Breakdown consists of the progressive microscopic degradation of a dielectric material when it is exposed to electrical stress. This stress results in: (i) breaking bonds in the dielectric (where oxygen vacancies commonly appear in metal oxides), and (ii) migration of ions from the adjacent electrodes into the dielectric. When the density of defects reaches a threshold, a conductive percolation path through the dielectric can be formed. In 3D oxides dielectric breakdown is more progressive for thinner dielectrics, while in 2D layered dielectrics it has been suggested to take place layer-by-layer^107,108^. However, this layer-by-layer breakdown has been only observed by conductive atomic force microscopy (CAFM), where the electric field is confined by a nanoscale tip, and should be confirmed for macroscopic devices.

It is worth noting that dielectric breakdown always takes place at the electrically weakest location of the dielectric. Preexisting defects can favor a wide range of unwanted charge transport phenomena, such as charge trapping and de-trapping (resulting in RTN), and after some time can trigger the formation of new defects in surrounding areas. At the device level this results in an increase of the leakage current called stress induced leakage current (SILC), which finally triggers dielectric breakdown. Therefore, the presence of local lattice distortions in 2D insulators is a very important source of non-idealities which can accelerate dielectric breakdown^109^. At the same time, the number of local defects in both 2D and 3D insulators depends strongly on the synthesis process used. In general, dielectric breakdown should be investigated with a statistically relevant experimental design.

### Impact of mechanical strain

One very promising direction for the application of 2D devices is flexible electronics^110^. Thus, the impact of mechanical strain on the device performance can be a limiting factor specific to these applications. Previous studies on MoS_2_ FETs with hBN suggest that the performance of these devices is not affected by mechanical strain up to 1.5%^11^. In contrast, devices with Al_2_O_3_ insulators exhibit a sizable threshold voltage shift for a strain of only ~0.07%, which can also be exploited for applications as piezoresistive strain sensors^111^. This is in line with experimental reports on the relatively high piezoresistive gauge factor of TMDs like MoS_2_^112^ or PtSe_2_^113^. It appears that a strong impact of strain on the performance of the devices with amorphous oxides is related to the strain changing the bandgap of MoS_2_^111^, which affects the relative alignment of the defect bands to the conduction/valence band edges, and may also shift the Fermi level. This in turn changes the charge state of border traps near the interface and the density of active defects. Thus, crystalline insulators which contain a lower amount of defects appear to be more suitable candidates also for flexible electronics applications.

## Future development of 2D electronics

Taking into account the current state-of-the-art discussed above, in the following we propose research strategies for the development of 2D devices with the three most important types of gate insulators, which are amorphous 3D oxides, native oxides of 2D semiconductors and crystalline insulators.

### Devices with conventional 3D oxides

The main limitations for integration of 3D oxides into electronic devices based on 2D materials result from their poor interface quality as well as their wide defect bands and their respective energetic alignment with the conduction/valence bands of the channel. It is important to realize that only a joint solution for both issues can be considered a promising way forward. For instance, recent attempts to passivate oxide interfaces by using thin molecular crystals^39,40^ improve only the interface quality, while deposition of these materials in homogeneous layers may present a technological challenge. However, it appears to us that a complete passivation of the defect bands would require thicker seeding layers which are incompatible with the sub-1 nm EOT requirement. Thus, we argue that in addition to further process optimization and van der Waals integration of the interfaces, an important goal of future research on 3D oxides for 2D devices should be the selection of the most favourably matched 2D channel/3D oxide combinations. Namely, for p-FETs it is important to select an insulator with defect bands energetically far from the valence band of the channel, while for n-FETs they must be separated from the conduction band. As a result, the density of active border traps being able to contribute to charge trapping will be reduced, thus leading to more stable device operation. The energetic alignment of defect bands can be extracted either by fitting TCAD models to measured stability characteristics of 2D devices^23,71^ or by using experimental methods such as the incremental hysteresis sweep method^50,93^ or trap spectroscopy methods previously developed for Si devices^99^.

**Fig. 6:**
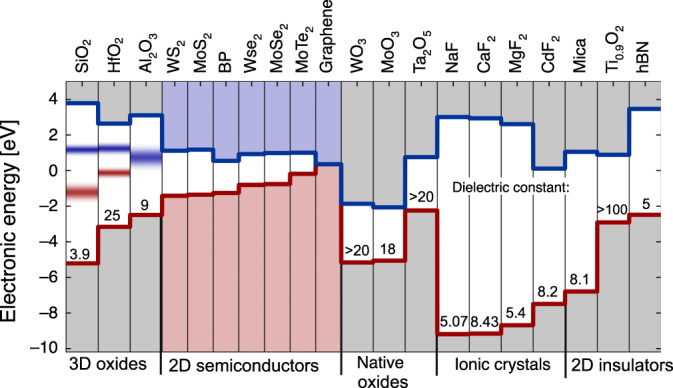
**Band diagram of different insulators matched with 2D semiconductors**. Left: energetic alignments of already known defect bands (gray areas) in several 3D oxides^23,93,99,114^ relative to the conduction and valence band edges of some frequently used 2D channel materials^157^ (center). Right: matching of potentially interesting native oxides, ionic crystals and 2D insulators with the same 2D channels. Note that HfO_2_ can be both a 3D oxide and the native oxide of HfS_2_ and HfSe_2_ and might have similar defect bands in both cases. The dielectric constants are given next to the valence band of the insulators while the numerical values are summarized in tabular form in Box 3. The zero energy would correspond with the Si midband.

For the most relevant oxides (SiO_2_, HfO_2_, Al_2_O_3_), defect bands have already been extracted for Si devices^99,114^ as well as for 2D FETs^23,71,93^ and reconfirmed for different technologies. In Fig. 6 we show the energetic alignments of known defect bands in oxides relative to the band edges of graphene, BP and the most commonly used TMD channel materials. While the defects responsible for these bands can be considered a fundamental property of the insulator, the parameters of the defect bands vary weakly with the processing conditions^114^. Even though some materials may contain additional but currently unknown defect bands, some competitive combinations of insulators and 2D channels (e.g. Al_2_O_3_ on BP for p-FETs and perhaps HfO_2_ on MoS_2_ for n-FETs) can be preselected based on Fig. 6. Furthermore, another degree of freedom is the adjustable number of layers in a 2D channel, which affects the bandgap and thus may allow tuning the relative alignment of the channel band edges and defect bands in the insulator.

Integrating 3D oxides with 2D materials by van der Waals forces can be achieved by passivating the 2D surfaces with molecular crystals. While this approach appears promising, it requires further investigations as this step may introduce additional limitations, such as poor dielectric strength of the seeding layer, insufficient barrier heights, low adhesion, and eventually a significant increase in EOT.

An alternative design route would be the creation of clean and stable oxide surfaces first, although in our opinion this is challenging for all previously discussed 3D oxides, in part due to their chemical reactivity. In theory, this problem can be solved by using an inverse design approach^115^, where the desired functionality (e.g. smooth interface) is declared first, and ab initio modeling is then performed to predict which stable and synthesizable materials would exhibit the required properties. These predictions can be based on genetic algorithms^116^ that identify materials with target properties while allowing deviations. In addition to predicting 3D oxides with smooth interfaces, we suggest that the same approach can be used to identify which new compounds will have the most suitable dielectric properties and targeted energetic alignments of defect bands. Finding ideal insulators therefore requires at least three coupled criteria, i.e. dielectric properties, interface quality and defect bands, and result in the required chemical formula. Once a new material is synthesized, extensive experimental characterization will still be required with respect to all important parameters. However, we expect the probability of finding 3D oxide compounds which would simultaneously satisfy all necessary requirements and could be easily synthesizable to be rather small. Instead, we propose a combination of theory and experiments to investigate alternative insulators for 2D electronics in the near future.

### Devices with native oxides of 2D materials

The use of native oxides of 2D semiconductors^41–44^ is a promising way to overcome the limitations of 3D oxides as they may lead to improved interface quality. Among the materials which have already been investigated as gate insulators in 2D devices, we mention Ta_2_O_5_, which can be thermally oxidized from TaS_2_^41^, and HfO_2_, which can be obtained by ambient exposure of HfSe_2_^42^ or by plasma oxidation of HfS_2_^43^. This field is relatively unexplored, and in many cases the oxidation of 2D semiconductors leads to the formation of non-stoichiometric metal oxides, such as HfO_*x*_ for HfS_2_^44^.

Nevertheless, we think that the integration of non-stoichiometric oxides into 2D devices could be of interest, as it allows tuning the charge carrier concentration in the channel through charge transfer doping, where the doping concentration can be varied by changing the oxygen content *x*^105,117^. Thus, it might be possible to vary the oxygen content of the native oxide using inverse design algorithms^115^ to possibly predict the compounds with desired functionality. One technological option is plasma oxidation of multi-layer WSe_2_ which leads to the formation of the native oxide WO_*x*_ and efficient p-doping of WSe_2_ FETs^118^. It has further been shown that WO_*x*_ and MoO_*x*_, a native oxide of MoS_2_, can be obtained by various oxidation methods without damaging the underlying 2D material^119,120^. However, the use of these native oxides as gate insulators in 2D devices has not been demonstrated so far. On the other hand, AlO_*x*_ encapsulation layers^121^ and HfO_*x*_ gate insulators^117^ have been used to dope MoS_2_ FETs, even though these oxides are not native for MoS_2_. The stoichiometric native oxides WO_3_ and MoO_3_ have been known for a long time^122,123^ and also appear promising for WSe_2_ and MoS_2_ FETs. While each 2D material normally has only one native oxide, some 2D semiconductors (e.g. MoS_2_) can be also matched with native oxides of other materials (e.g. Ta_2_O_5_)^41^. However, recent studies suggest that some of these materials, as well as Ta_2_O_5_, have rather narrow bandgaps^124^, and exhibit unfavourable band offsets with such widely used 2D semiconductors as MoS_2_ (Fig. 6).

In addition, these native 2D oxides are still amorphous and will therefore contain distinct defect bands. Furthermore, the non-stoichiometric ones are expected to exhibit very high defect densities and limited dielectric strength^105^. Thus, as well as for 3D oxides, we suggest that decreasing the number of active defects will present a challenge, and the locations of the defect bands are currently unknown for most of these materials.

Overall, the true potential of native oxides for 2D devices remains to be explored and the research in this field is now in an early stage. While theoretical predictions may provide some guidelines, we suggest that many technological issues remain with respect to fully scalable integration and controllable quality, as there is currently no clear recipe on how to achieve the ultimate goal of minimized or even completely eliminated defect bands. Furthermore, all native oxides except HfO_2_ do not seem to be competitive with respect to their dielectric properties, which could make them applicable only as passivation layers^124^. Thus, in the following section we discuss insulators which could retain their crystalline structure even when fabricated as ultra-thin layers.

Box 3 Alignments of known defect bands in oxides In the table in Box 3 we summarize the band gaps, band offsets and the parameters of defect bands for SiO_2_, HfO_2_ and Al_2_O_3_. In SiO_2_ and HfO_2_ two defect bands are typically used, while for Al_2_O_3_ only one defect band is known. It is important that the values for SiO_2_ have been verified for both 2D FETs^23,93^ and Si technologies^99,114^. For other materials no information about defect bands is available. However, it is thought that in crystalline insulators the width of the defect band would be much smaller.

The table below summarizes dielectric parameters of several oxide insulators and exact alignments of known defect bands^93,114^. The widths of the defect bands depend more on the material quality than their energetic alignments. They are typically larger in 2D technologies with lower quality oxides.

### Devices with crystalline insulators

Crystalline materials theoretically provide the largest potential for obtaining defect-free insulators and overcoming problems associated with both interface quality and defect bands. The most promising results for 2D FETs have recently been obtained for crystalline layered 2D insulators, in particular hBN^108,125^ and mica^126^, as well as the ionic crystal CaF_2_^46,62,127,128^. These materials and some other known fluorides are summarized in Fig. 6.

The most widely studied crystalline insulator for 2D materials is hBN^11,45,54,58^. Devices using hBN typically exhibit a sizable improvement in terms of *SS*^54^ and mobility^11^ and also show considerably reduced charge trapping compared to 3D oxides^45,58^. This can be explained by the well-defined surface of hBN and the low density of border traps in this crystalline material. Unfortunately, hBN has mediocre dielectric properties, such as a rather narrow bandgap of about 6 eV^129^, a small dielectric constant of 5.06^130^, and unfavourable band offsets to most 2D materials (see Fig.6). As scaled FET technologies require an EOT below 1 nm (corresponding to a physical hBN thickness of below 1.3 nm), hBN is expected to result in excessive thermionic and direct tunneling leakage currents potentially orders of magnitude larger than those expected for high-k oxides. However, the exact nature of leakage currents across monolayer and multilayer hBN as well as the impact of the van der Waals gap is still not fully understood. As an example, it has been recently reported^68^ that Ti/hBN(5–6 nm)/Au structures exhibit leakage currents surprisingly lower than much thicker Pt/SiO_*x*_N_*y*_:Ag(50 nm)/Pt^131^ structures.

Knowledge regarding the dielectric strength of hBN as well as its breakdown mechanisms is also very scarce. Previous studies have been conducted mostly at the material-level^107,108^ and metal-insulator-metal device configurations (e.g. memristors^132^). It has been shown that thick (>10 nm) multilayer hBN stacks obtained by mechanical exfoliation experience a layer-by-layer breakdown^107,108^ when exposed to high electric fields. However, at the device level native defects in hBN, such as nitrogen and boron vacancies and antisites predicted by DFT modeling^88,89^, are expected to mask this phenomenon, leading to a more progressive breakdown process^109^.

More experiments are required to clarify the dielectric properties and the breakdown behavior of hBN stacks in FETs to fully understand the potential of hBN for ultra-scaled digital 2D devices. Nevertheless, it can already be concluded that hBN is indeed a promising insulator for analog 2D devices that do not require aggressive thickness scaling and operate at low electric fields, such as photodetectors^35^ and sensors^36^ employing graphene channels.

Mica is another interesting layered 2D insulator that has been investigated as a back-gate insulator in GFETs^126^ and top-gate insulator in MoS_2_ FETs^133^. In addition to having a well-defined surface, this 2D insulator has a reasonably high permittivity (8.1) and a wide bandgap (10.5 eV), which would address some of the limitations of hBN. However, the studies performed on exfoliated flakes do not yet allow to assess the real potential of mica. It is worth noting that mica may be used as a growth substrate for other 2D materials, and thus lend itself to future integration schemes^134^. Other potentially interesting 2D insulators include crystalline Ti_0.9_O_2_^135^, 2D silicon dioxide^136^ and other atomically thin oxides^137^, although all these materials are still far from being integrable with conventional silicon technology.

3D ionic crystals possess well-defined surfaces and are hence discussed here. In our opinion, the most promising candidates for applications as gate insulators are epitaxial fluorides which form a wide class of different insulators and other emerging materials^138^. Many fluoride insulators have competitive dielectric properties, chemically inert surfaces, low density of insulator defects and high electric stability, which makes them highly suitable for 2D electronics. Recently it has been shown that competitive MoS_2_ FETs can be created using epitaxial CaF_2_ insulators of only 2 nm thickness^46^. Such thicknesses are currently barely achievable with 3D oxides owing to their poor amorphous quality. Remarkably, CaF_2_ is also competitive with high-k oxides in terms of its dielectric properties (e.g. wide bandgap of 12.1 eV and reasonably high dielectric constant of 8.43). In addition, it has been known for some time that CaF_2_ can form a well-defined van der Waals interface with 2D channels^47^. This feature allows heteroepitaxy of 2D materials on CaF_2_(111)^83,84^, thus opening additional opportunities for the creation of scalable 2D devices. Owing to the high crystalline quality of CaF_2_ insulators, MoS_2_ FETs on CaF_2_/Si(111) substrates were found to be extremely stable with respect to charge trapping^46^. At the same time, the analysis of breakdown in CaF_2_ using CAFM suggests that this material is highly homogeneous and has a very high dielectric strength.

These promising early results lead us to conclude that close attention should be paid also to other fluoride insulators such as LaF_3_, MgF_2_, BaF_2_, SrF_2_, and many others. Similarly to CaF_2_, many of these materials have very wide bandgaps (e.g. 11.4 eV for SrF_2_ and 13 eV for MgF_2_) but at the same time mediocre dielectric constants (e.g. 6.4 for SrF_2_ and 5.4 for MgF_2_). Nonetheless, for CaF_2_ theoretically predicted (Fig.3b) and measured (Fig.3c) leakage currents are still comparable to high-k oxides like HfO_2_, since the band offsets to Si and 2D semiconductors are high. However, for other fluorides possible limitations which may arise from their relatively low dielectric constants still have to be understood by performing electrical characterization of thin layers. Apart from 2D FETs, ionic crystals can be also of interest for analog 2D devices and other applications. For instance, the performance of 2D-based photodetectors^35^ strongly depends on the insulator properties and can be adjusted by choosing appropriate insulators. Also, the exciton properties (e.g. radiative lifetime) in excitonic devices^139^ strongly depend on the thickness and type of the insulator matched with the 2D material. However, at the present early stage of research it is not possible to make a final conclusion on the future potential of ionic fluoride crystals as their fundamental properties (e.g. chemical structure and breakdown mechanisms) are not well understood and thus require further in-depth studies. Furthermore, the compatibility of positive ions such as Ca^2+^ or Mg^2+^ with CMOS and possibly future beyond-CMOS technologies will have to be assessed by the community.

In addition to their use as insulators, the wide class of epitaxial fluorides^138^ also contains numerous materials with other fascinating properties. These are, for instance, antiferromagnetic NiF_2_^140^ and MnF_2_^141^, diamagnetic ZrF_2_^141^ and ferroelectric BaMgF_4_^142^. One of the most promising research directions is the use of ferroelectric BaMgF_4_^142^ in steep slope devices such as negative capacitance (NC) FETs, which could be game-changers for future low-power electronics. Previously reported NC FETs with MoS_2_ employed hafnium zirconium oxide (HZO)^32,33^, polymers^143^ and layered CuInP_2_S_6_ (CIPS)^144^ ferroelectrics. The best *SS* reported for devices with HZO approach 6 mV/dec at room temperature^32^. Although HZO appears more technologically relevant because of its CMOS compatibility and large area fabrication possibilities, we expect that some of these devices (e.g. HZO/Al_2_O_3_/MoS_2_ NC-FETs) will likely face the same problems with poor quality interfaces and border traps as standard 2D FETs. Thus, it is tempting to project that the use of BaMgF_4_, which has already been applied in ferroelectric Si-based devices^145^, will allow overcoming already achieved *SS* values while also leading to improved device stability. As a final comment, it is worth pointing out that the potential of NC-FET technologies is currently under debate by the community, since some studies suggest that the NC effect would not benefit devices that already have strong electrostatics, such as 2D FETs^146^.

Overall, it appears to us that crystalline insulators are currently the most promising materials for applications in various devices based on 2D materials, since their possible physical limitations appear easier to address than in amorphous oxides. Even at the present stage of research, the technological limitations of the currently used insulators are apparent. For instance, most devices with hBN and other layered 2D insulators employ tens of nanometers thick layers deposited by mechanical exfoliation. At the same time, attempts at scalable growth of hBN using CVD^147^ and MBE^148^ have not resulted in superior device performance than conventional 3D oxides^149^. Thus, fully scalable methods to grow layered 2D insulators have to be further developed. In the case of hBN, this currently involves temperatures above 800 °C^150^, which is far above the maximum allowed by the thermal budget of CMOS technologies (about 450 °C) for back end of line integration^151^. In contrast, for CaF_2_ and some other related insulators fully scalable MBE growth techniques partially exist, while the optimal growth temperatures for a few nanometer thick layers on Si appear more reasonable. For instance, MBE growth of CaF_2_ films at 250 °C results in high crystalline quality and pinhole-free layers^46,152^. Also, MBE growth of 2D semiconductors (e.g. MoSe_2_ and MoTe_2_) on CaF_2_(111) is possible at temperatures below 400 °C^83,84^. Furthermore, it is possible that heteroepitaxy of CaF_2_ and other related insulators (e.g. SrF_2_, MgF_2_, BaF_2_) on top of 2D semiconductors will be also possible at moderate growth temperatures. While for now a considerable limitation of ionic crystals is that they have been used only as a back-gate insulators^46^, we think that the latter requires more attention as this would allow obtaining Si/fluoride/2D/fluoride heterostructures as required for top-gated 2D FETs which is essential for integrated circuits.

## Conclusions and outlook

Since 2004, when the field effect in graphene was reported for the first time^153^, many devices with different 2D materials have been demonstrated. The most prevalent among them are FETs with semiconducting 2D channels, which could be important building blocks for future post-silicon electronics. Owing to the thin body of 2D materials, their use as channels in FETs would allow suppressing the well-known short-channel effect of Si transistors, thus opening a route towards sub-5 nm device dimensions and prolonging Moore’s law^154^. Despite these high expectations and the progress made thus far, over a decade of intensive research has not led to a commercial 2D device technology^155,156^. One important reason discussed here is the lack of insulators suitable for integration into fully scalable 2D process flows, which would enable a competitive device performance and stability.

Most 2D FET prototypes reported in literature have been made using tens of nanometers thick oxide insulators, with no clear strategy on how to scale them down to sub-1 nm EOT as required for commercially competitive FETs. Thus, we firmly believe that the primary challenge is to identify fully scalable insulators for 2D FETs and to collect in-depth information about their properties. When considering a certain insulator as a potential candidate for integration into 2D technologies, its dielectric properties have to be identified first, as they are important for low gate leakage currents in scaled devices. Then, information about the quality of the interface between the insulator and 2D materials has to be gathered. This is required because a large amount of interface states, such as oxide dangling bonds, will result in poor device performance. Finally, attention has to be paid to the location and density of its defect bands, as well as their energetic alignment to various channel materials and possible mechanisms for the creation of new insulator defects under electric stress. This information is essential for further improving the stability and dielectric strength of 2D FETs. This is an important aspect, because although considerable progress has been made in optimizing 2D device performance, the stability of 2D FETs and analog devices is far from being competitive with Si technologies and remains poorly understood.

2D devices and their technology face enormous challenges towards commercial uptake, and we have identified the search for a perfectly matching insulator/semiconductor combination as particularly urgent. This is because from the myriad of possible material combinations the right choice has to be made at as early a stage as possible by considering their scaling potential. Based on this observation, several new research problems can be formulated. First of all, research on high-k oxides as potentially interesting insulators for 2D devices should be continued, even though at present the problem of how to fabricate a clean interface to 2D materials presents an enormous challenge. Here, efforts related to improving the interface quality of 3D oxides by rapid thermal annealing^38^ and further development of native oxides^41–44^ of 2D semiconductors should be continued. Also, the correlation between growth conditions and their fundamental defect bands must be further explored. Next, we feel that two alternative directions for 2D FET technologies appear promising: The first alternative is the use of layered 2D insulators which produce near-perfect interfaces with 2D channels. One of these materials is hBN, but more investigations are required to clarify several unique properties of this dielectric, such as plane-to-plane interactions and electron tunneling across van der Waals structures. We further hope that this review triggers a more intensive search for other 2D insulators, such as mica and 2D oxide nanosheets. The second alternative is the use of ionic crystals such as fluorides, which create near-perfect van der Waals interfaces with 2D channels and simultaneously have good dielectric properties. Up to now, only CaF_2_ has been used as an insulator for 2D FETs^46^. However, the recent demonstration of epitaxial growth of 2D materials on CaF_2_^83,84^ has opened a potential route to very large scale integration and perhaps to the development of 2D FETs based on fluoride/2D/fluoride heterostructures. Furthermore, there are many fluorides beyond CaF_2_ with fascinating properties as insulators or as magnetic and ferroelectric materials, which should in future allow to create more competitive 2D devices beyond FETs.

To conclude, the identification of the best insulators for 2D electronics presents an important roadblock of modern nanoscience and the apparent lack of information related to this problem should no longer be ignored. We are confident that further development of this research topic will sooner or later enable 2D electronics for commercial applications.
